# A Remarkable Epitaph

**Published:** 1902-12-20

**Authors:** Algernon Ashton


A REMARKABLE EPITAPH.

Sir,—The other day, after an absence of 23 years, I once more happened to visit the highly-interesting old Bunhill Fields Burial Ground. It is situated in the City Road, just opposite the house where John Wesley lived and died, and contains, among other memorable tombs, the graves of John Bunyan, Daniel Defoe, and Isaac Watts. But there is one monument upon which is inscribed so curious and remarkable an epitaph that I cannot refrain from quoting it:—

South Side.

Here lyes Dame Mary Page,
Relict of Sir Gregory Page, Bart.,
She departed this life March 11, 1728,
In the 56 year of her age.

North Side.

In 67 months she was tap'd 66 times,
Had taken away 240 gallons of water,
Without ever repining at her case,
Or ever fearing the operation.

I am, etc.,

Algernon Ashton.

